# Specialist learning curves and clinical feasibility of introducing a new MRI grading system for skeletal maturity

**DOI:** 10.1093/bjro/tzae008

**Published:** 2024-04-10

**Authors:** Francesca De Luca, Thröstur Finnbogason, Ola Kvist

**Affiliations:** Department of Clinical Neuroscience, Karolinska Institute, Tomtebodavägen 18 a, 171 77 Stockholm, Sweden; Department of Radiology, Karolinska University Hospital, Eugeniavägen 3, 171 64, Stockholm, Sweden; Department of Pediatric Radiology, Karolinska University Hospital, Eugeniavägen 23, 171 64, Stockholm, Sweden; Department of Pediatric Radiology, Karolinska University Hospital, Eugeniavägen 23, 171 64, Stockholm, Sweden; Department of Women’s and Children’s Health, Karolinska Institute, Tomtebodavägen 18a, 171 77, Stockholm, Sweden

**Keywords:** growth plate, skeletal maturity, magnetic resonance imaging, MRI-based assessment of skeletal maturity, learning curve

## Abstract

**Objective:**

MRI is an emerging imaging modality to assess skeletal maturity. This study aimed to chart the learning curves of paediatric radiologists when using an unfamiliar MRI grading system of skeletal maturity and to assess the clinical feasibility of implementing said system.

**Methods:**

958 healthy paediatric volunteers were prospectively included in a dual-facility study. Each subject underwent a conventional MRI scan at 1.5 T. To perform the image reading, the participants were grouped into five subsets (subsets 1-5) of equal size (*n*∼192) in chronological order for scan acquisition. Two paediatric radiologists (R1-2) with different levels of MRI experience, both of whom were previously unfamiliar with the study’s MRI grading system, independently evaluated the subsets to assess skeletal maturity in five different growth plate locations. Congruent cases at blinded reading established the consensus reading. For discrepant cases, the consensus reading was obtained through an unblinded reading by a third paediatric radiologist (R3), also unfamiliar with the MRI grading system. Further, R1 performed a second blinded image reading for all included subjects with a memory wash-out of 180 days. Weighted Cohen kappa was used to assess interreader reliability (R1 vs consensus; R2 vs consensus) at non-cumulative and cumulative time points, as well as interreader (R1 vs R2) and intrareader (R1 vs R1) reliability at non-cumulative time points.

**Results:**

Mean weighted Cohen kappa values for each pair of blinded readers compared to consensus reading (interreader reliability, R1-2 vs consensus) were ≥0.85, showing a strong to almost perfect interreader agreement at both non-cumulative and cumulative time points and in all growth plate locations. Weighted Cohen kappa values for interreader (R1 vs R2) and intrareader reliability (R1 vs R1) were ≥0.72 at non-cumulative time points, with values ≥0.82 at subset 5.

**Conclusions:**

Paediatric radiologists’ clinical confidence when introduced to a new MRI grading system for skeletal maturity was high from the outset of their learning curve, despite the radiologists’ varying levels of work experience with MRI assessment. The MRI grading system for skeletal maturity investigated in this study is a robust clinical method when used by paediatric radiologists and can be used in clinical practice.

**Advances in knowledge:**

Radiologists with fellowship training in paediatric radiology experienced no learning curve progress when introduced to a new MRI grading system for skeletal maturity and achieved desirable agreement from the first time point of the learning curve. The robustness of the investigated MRI grading system was not affected by the earlier different levels of MRI experience among the readers.

## Introduction

The growth plate is the section of cartilage in the long bones where bone formation occurs, located between the epiphysis and metaphysis of long bones in children and adolescents. Out of the three growth plate cartilage layers, the zone of provisional calcification in the hypertrophic zone is the front of calcification in the vicinity of the metaphysis.^1^ Radiological evaluation of the degree of closure of the growth plate is considered essential in various clinical applications, such as assessment of skeletal maturity,^2^ diagnosis and treatment of developmental and growth disorders,^3^^,^^4^ and athlete age assessment in competitive sports.^5–7^

Among the currently used imaging modalities to assess skeletal maturity, MRI has played an emerging role due to its ability to depict the skeletal maturity process by evaluating the layers of growth plate cartilage.^8^ MRI has an advantage compared to the more broadly used radiography and CT, as radiography and CT can only depict the appearance of bone mineralisation. In contrast, MRI detects gradual changes in fluid distribution that accompany the mineralisation process. Unlike radiography and CT, MRI does not entail exposure to ionizing radiation, which is preferable when assessing skeletal maturity in the paediatric population.^9^ Despite the advantages mentioned above, the use of MRI to assess skeletal maturity is still relatively limited in radiological practice. MRI images of the growth plate show a level of detail closer to histopathology than radiology, which renders the interpretation of MRI images at the growth plate challenging even for experienced radiologists. Several MRI grading systems for skeletal maturity have been proposed in the literature, using different grading stages and growth plate locations.^10–15^ Despite these efforts, there is still a knowledge gap about the impact of radiologists’ earlier MRI experience and the clinical feasibility of these MRI grading systems to assess skeletal maturity. This study aimed to chart the learning curves of paediatric radiologists when using an unfamiliar MRI grading system of skeletal maturity and to assess the clinical feasibility of implementing said system.

## Methods

### Participants

Healthy paediatric volunteers were prospectively included in a dual-facility study (censored and censored) for a conventional MRI between May 2017 and April 2018. The same cohort was included in a previously published study evaluating the correlation between chronological age, body mass index, physical activity, and skeletal maturation at the time of imaging.^16^ Inclusion criteria were: verified birth certificate from the respective national authority. Exclusion criteria were: volunteers' residency >6 months outside the country conducting the study, verified history of bilateral trauma in the vicinity of the growth plate, clinically verified chronic disease, long-term therapy affecting the growth plate, previous or current pregnancy indicated by pregnancy screening for all female subjects, and incomplete MRI examination. The association between age and maturity using the investigated MRI grading system for skeletal maturity in the population has been documented in a previously published study.^16^ The current investigation focused on the learning curves of paediatric radiologists previously unfamiliar with the MRI grading system of skeletal maturity used in the study. The local ethics committee approved the study (ethical approval number 2017/4-31/4), which was performed according to the Declaration of Helsinki. Written consent/assent according to the ethical guidelines was obtained from all volunteers or legal guardians.

### Study population

Nine hundred fifty-eight healthy paediatric volunteers were included in the study. Detailed information about the study population demographics can be found in Table 1. Stages 1 and 2 were not seen in any participant due to the fact that the population consisted of individuals older than 13.0 years of age.

**Table 1.** Patients’ characteristics.

	Sex	Man	
		*n*	Mean age, years (SD), age range, years
**Learning curve time point**	Subset 1	115	17.6 (2.2), 14-21
	Subset 2	97	17.0 (2.0), 14-21
	Subset 3	79	17.7 (2.4), 14-21
	Subset 4	83	17.3 (2.3), 14-21
	Subset 5	103	18.6 (2.3), 14-21
	Total count n (%)	477 (50%)	

	**Sex**	**Female**	
		
		*n*	Mean age, years (SD), age range, years

	Subset 1	76	18.2 (2.1), 14-21
	Subset 2	94	16.9 (2.3), 14-21
	Subset 3	113	17.6 (2.4), 14-21
	Subset 4	108	17.4 (2.1), 14-21
	Subset 5	90	18.4 (2.2), 14-21
	Total count n (%)	481 (50%)	

### MRI acquisition

The MRI scans were obtained with a 1.5 T conventional MRI scanner using a 2D spoiled gradient multi-echo sequence with a magnetization transfer saturation pulse. These sequences, in combination with the long-term averaging technique, were specially designed for neck and cervical spine imaging to suppress pulsation artefacts. Scan acquisitions for all paediatric volunteers included MRI of five different growth plate locations, namely distal radius, distal femur, proximal and distal tibia, and calcaneus. The non-dominant side of the volunteer was imaged unless there was a known history of trauma near the growth plate, in which case the dominant side was preferred. All MRI scans were obtained within six months of the volunteers’ most recent birthday. All anatomical regions were examined simultaneously in each subject with a single MRI scan acquisition with dedicated extremity coils. (detailed information about the MRI study protocols can be found in Supplementary Table S1).

### Image analysis

**Figure 1.**
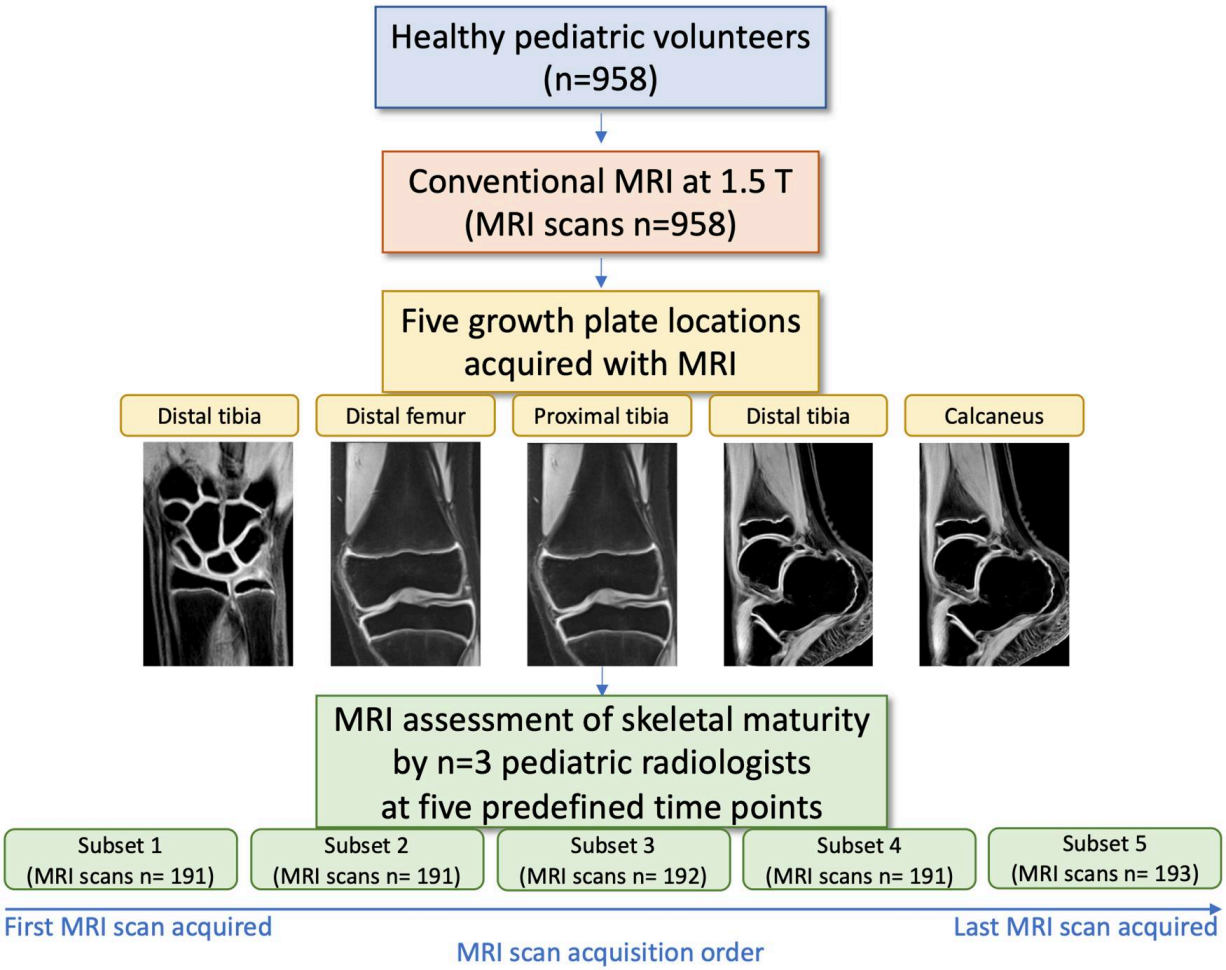
Study workflow. * In case of discrepancies, an unblinded consensus was obtained by a third paediatric radiologist.

**Figure 2.**
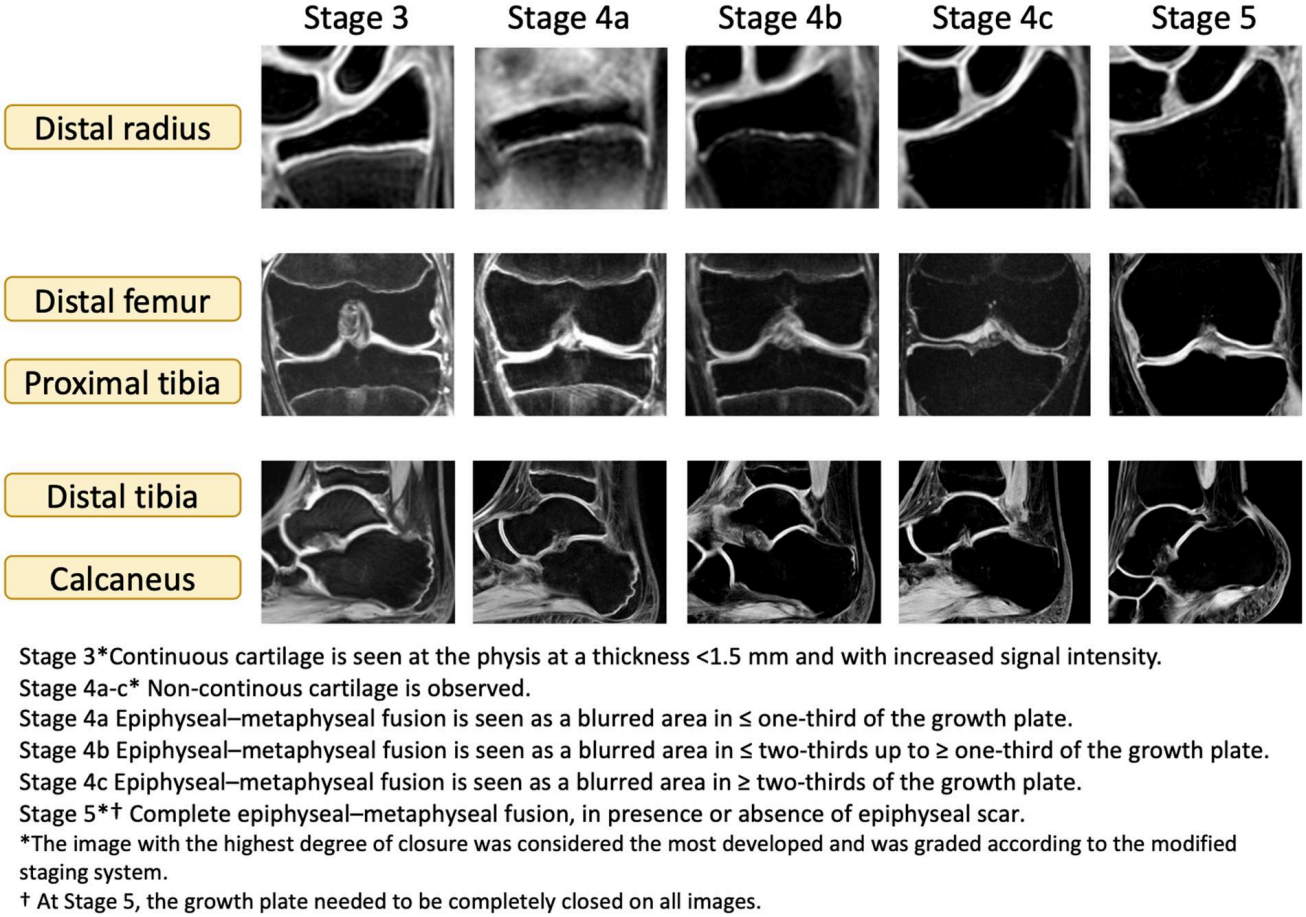
MRI images with the related MRI grading system based on the modified version of Kellinghaus’^17^ and Dedouit et al.^11^

All MRI scans were independently evaluated by two paediatric radiologists with varying levels of MRI experience (reader 1, R1: 25 years’ experience; reader 2: R2, three years’ experience), both of whom were previously unfamiliar with the MRI grading system of skeletal maturity investigated in this study. The readers were blinded from all clinical and demographic information. No instructions about the use of the investigated MRI grading system of skeletal maturity were given prior to grading. The images were evaluated in the local PACS system (picture archive and communicating system). To perform the image reading, the participants were grouped into five subsets (subsets 1-5) of equal size (n∼192) in chronological order for scan acquisition, ie, group 1 was the first 191 imaged participants, and group 5 was the last 193 (Figure 1). The age range was the same for all subsets (Table 1). The MRI grading system used in the study was created from a modified version of Kellinghaus^17^ and Dedouit et al^11^ and included 7 different stages: stages 1-5, including three substages of stage 4 (stages 4a-c). Coronal views were used to grade the skeletal maturity at the distal radius, distal femur, and proximal tibia. In contrast, sagittal views were used for grading at the distal tibia and calcaneus. The image with the highest grade of closure was considered the most developed and was graded according to the modified staging system (detailed information about the MRI grading system can be found in Figure 2 and a more detailed description in Supplementary Table S2). Concordant evaluations between the two blinded readers established the consensus reading (R1+R2). In cases of disagreement, an unblinded consensus was obtained by a third reader (reader 3, R3), a paediatric radiologist with 13 years of MRI experience, also new to the MRI grading system investigated in this study. Further, R1 performed a second blinded image reading for all included subjects with a memory wash-out of 180 days.

### Statistical analysis

Weighted Cohen kappa^18^ was used to evaluate the inter-reader reliability (R1 vs consensus; R2 vs consensus) at each time point (subset 1, 2, 3, 4, 5). The Kappa values were interpreted as follows: no agreement ≤0.20; minimal agreement = 0.21-0.39; weak agreement = 0.40-0.59; moderate agreement = 0.60-0.79; strong agreement = 0.80-0.90; almost perfect agreement <0.9.

To evaluate the cumulative effect of the learning process, the same analysis was also performed at cumulative time points (subset 1, subset 1 + 2, subsets 1-3, subsets 1-4, and subsets 1-5). Weighted Cohen kappa^18^ (ref) was also used to evaluate the inter-reader (R1 vs R2) and intra-reader reliability (R1 vs R1) at non-cumulative time points. Statistical analysis was performed with Statistical Package for the Social Sciences (SPSS, IBM Corp., Armonk, NY).

## Results

### Reliability using the MRI grading system of skeletal maturity

Weighted kappa values (CI 95%) for each pair of blinded readers compared to consensus reading (R1-2 vs consensus) for non-cumulative and cumulative time points are shown in Tables 2 and 3. A strong to almost perfect inter-reader reliability was found at all non-cumulative time points in all growth plate locations (mean kappa R1-2 vs consensus: ≥0.85 distal radius and distal femur, ≥0.87 proximal tibia, ≥0.86 distal tibia, ≥0.90 calcaneus). Also for cumulative time points, a strong to almost perfect inter-reader reliability was seen in all growth plate locations (mean kappa R1-2 vs consensus: ≥0.88 distal radius and distal femur, ≥0.86 distal tibia, ≥0.90 proximal tibia and calcaneus). Weighted Cohen kappa values for inter-reader (R1 vs R2) and intra-reader reliability (R1 vs R1) were ≥0.72 at non-cumulative time points, with values ≥0.82 at subset 5, Supplementary Tables S3 and S4. These results suggest that the blinded readers had good clinical confidence using the MRI grading system from the first time point of the learning curve, despite having different work experience and without any prior experience using the MRI grading system of skeletal maturity investigated in the study.

**Table 2.** R1–2 vs consensus, non-cumulative time points.

Learning curve phase	1		2		3		4		5	
	*Κ*	95% CI	*Κ*	95% CI	*Κ*	95% CI	*Κ*	95% CI	*Κ*	95% CI
**Distal radius**										
R1—consensus	0.90	0.85-0.94	0.81	0.76-0.85	0.89	0.85-0.93	0.94	0.90-0.97	0.91	0.86-0.96
R2—consensus	0.93	0.90-0.96	0.89	0.84-0.93	0.90	0.86-0.94	0.95	0.92-0.98	0.91	0.87-0.96
Mean kappa R1-2	0.92		0.85		0.90		0.95		0.91	
**Distal femur**										
R1—consensus	0.93	0.89-0.97	0.81	0.76-0.86	0.78	0.72-0.84	0.88	0.83-0.93	0.92	0.87-0.97
R2—consensus	0.92	0.89-0.96	0.90	0.86-0.94	0.92	0.88-0.96	0.88	0.82-0.94	0.90	0.84-0.96
Mean kappa R1–2	0.93		0.86		0.85		0.88		0.91	
**Proximal tibia**										
R1—consensus	0.90	0.85-0.95	0.85	0.81-0.90	0.88	0.83-0.93	0.94	0.89-0.98	0.99	0.96-1.01
R2—consensus	0.97	0.94-0.99	0.88	0.83-0.92	0.87	0.81-0.93	0.89	0.84-0.94	1	1
Mean kappa R1–2	0.94		0.87		0.88		0.92		1	
**Distal tibia**										
R1—consensus	0.88	0.82-0.94	0.87	0.81-0.92	0.89	0.83-0.94	0.95	0.90-0.99	0.90	0.82-0.98
R2—consensus	0.84	0.77-0.91	0.91	0.86-0.97	0.92	0.86-0.98	0.90	0.83-0.97	0.98	0.94-1.02
Mean kappa R1-2	0.86		0.89		0.91		0.93		0.94	
**Calcaneus**										
R1—consensus	0.87	0.79-0.96	0.88	0.82-0.94	0.93	0.87-0.99	0.90	0.84-0.97	0.92	0.84-1.00
R2—consensus	0.93	0.85-1.01	0.95	0.91-0.99	0.99	0.96-1.01	0.91	0.83-0.98	0.98	0.93-1.02
Mean kappa R1-2	0.90		0.92		0.96		0.91		0.95	
**Overall mean kappa**	0.91		0.88		0.90		0.91		0.94	

*Κ* = Kappa value*,* 95% CI = Confidence interval.

**Table 3.** R1–2 vs consensus, cumulative time points.

Learning curve phase	1		1–2		1–3		1–4		1–5	
	*Κ*	95% CI	*Κ*	95% CI	*Κ*	95% CI	*Κ*	95% CI	*Κ*	95% CI
**Distal radius**										
R1—consensus	0.90	0.85-0.94	0.85	0.82-0.88	0.87	0.84-0.89	0.88	0.86-0.90	0.89	0.87-0.91
R2—consensus	0.93	0.90-0.96	0.91	0.88-0.94	0.91	0.89-0.93	0.92	0.90-0.94	0.92	0.90-0.94
Mean kappa R1-2	0.92		0.88		0.89		0.90		0.91	
**Distal femur**										
R1—consensus	0.93	0.89-0.97	0.87	0.84-0.90	0.84	0.81-0.87	0.85	0.83-0.88	0.86	0.84-0.88
R2—consensus	0.92	0.89-0.96	0.91	0.88-0.94	0.91	0.89-0.94	0.91	0.89-0.93	0.91	0.89-0.93
Mean kappa R1-2	0.93		0.89		0.88		0.88		0.89	
**Proximal tibia**										
R1—consensus	0.90	0.85-0.95	0.88	0.84-0.91	0.88	0.85-0.91	0.89	0.87-0.91	0.90	0.88-0.92
R2—consensus	0.97	0.94-0.99	0.92	0.90-0.95	0.91	0.88-0.93	0.91	0.88-0.93	0.92	0.90-0.94
Mean kappa R1-2	0.94		0.90		0.90		0.90		0.91	
**Distal tibia**										
R1—consensus	0.88	0.82-0.94	0.87	0.83-0.91	0.88	0.84-0.91	0.89	0.86-0.92	0.89	0.87-0.92
R2—consensus	0.84	0.77-0.91	0.88	0.83-0.92	0.89	0.85-0.92	0.89	0.86-0.92	0.90	0.87-0.93
Mean kappa R1-2	0.86		0.88		0.89		0.89		0.90	
**Calcaneus**										
R1—consensus	0.87	0.79-0.96	0.88	0.83-0.93	0.89	0.85-0.93	0.89	0.86-0.93	0.90	0.87-0.93
R2—consensus	0.93	0.85-1.01	0.94	0.90-0.98	0.96	0.93-0.99	0.95	0.92-0.97	0.95	0.92-0.98
Mean kappa R1-2	0.90		0.91		0.93		0.92		0.93	
**Overall mean kappa**	0.91		0.89		0.90		0.90		0.90	

*Κ* = Kappa value*,* 95% CI = Confidence interval.

## Discussion

MRI has recently emerged as a non-invasive imaging method to assess the degree of skeletal maturity, given its ability to depict high-level details at the growth plate in the absence of ionizing radiation. Despite several existing MRI grading systems of skeletal maturity, there is still a knowledge gap about the impact of radiologists’ earlier MRI experience and the clinical feasibility of implementing these MRI grading systems of skeletal maturity. In this prospective study, we found the learning curves of two paediatric radiologists with different levels of MRI experience and first using an MRI grading system of skeletal maturity. The results from this study showed a strong to almost perfect inter-reader agreement among the readers regardless of the wide age range of included volunteers. Benefits from our results include reduced patient exposure to ionizing radiation-based modalities such as X-ray and CT to assess skeletal maturity in children, the applicability of the MRI grading system in a wide age range, and the generalizability of the investigated MRI grading system across paediatric radiologists without prior experience to the system.

In our study, the MRI grading system for skeletal maturity was investigated at five growth plate locations, including distal radius, distal femur, proximal and distal tibia, and calcaneus. Previous studies have evaluated MRI grading systems of skeletal maturity at a single growth plate location, such as the wrist,^10^ knee,^11–13^ or ankle.^14^^,^^15^ These studies reported an inter-reader agreement among readers ranging from moderate–good^10^^,^^11^ to strong–almost perfect.^8^^,^^12–15^ Accordingly in this study, a strong to almost perfect inter-reader agreement was found among the blinded readers at all growth plate locations. The applicability of the investigated MRI grading system to multiple growth plate locations strengthens the generalizability of our results. Further, the readers in our study did not have any earlier experience using the investigated MRI grading system. It is unclear in earlier studies how novel MRI grading systems were to the readers. Thus, it is unknown how reliably these MRI grading systems can be implemented in clinical practice.

The MRI grading system in this study included seven different stages, including three substages for stage 4. On the contrary, some of the previous studies investigated less detailed MRI grading systems of skeletal maturity, with a three-stage,^15^ five-stage,^11^ and six-stage^13^ MRI grading systems. Despite the use of less detailed MRI grading systems, no significantly higher inter-reader agreement was seen in these studies compared to the present work. Therefore, we can conclude that the MRI grading system used in this study results in more detailed information about the growth plate closure while preserving high inter-reader agreement. Another benefit of this study is the prospective nature of the study design. In contrast, some of the previous studies in the field of MRI grading systems of skeletal maturity were retrospective.^15^^,^^19^

In this work, learning curves were used in order to assess the time required for paediatric radiologists to acquire competence and proficiency in using the investigated MRI grading system of skeletal maturity. Despite the learning curve being a known powerful tool in medical education, few previous studies have investigated the role of the learning curve in the field of Radiology, and even fewer have evaluated learning curves among radiologists with regard to musculoskeletal imaging.^20–25^ To our knowledge, this is the first study evaluating the learning curve across paediatric radiologists when introduced to an unfamiliar MRI grading system of skeletal maturity.

The MRI assessment of skeletal maturity was performed by two blinded readers, a junior paediatric radiologist, and a senior paediatric radiologist. The readers achieved a desirable inter-reader and intra-reader agreement from the first time point of their learning curve. As a direct consequence, no significant improvement in their performance was seen over time or by cumulative cases investigated. Up to almost perfect agreement among the blinded readers was obtained regardless of their different levels of work experience, which strengthens the generalizability, clinical feasibility, and robustness of the investigated MRI grading system of skeletal maturity. The association between age and maturity using the investigated MRI grading system for skeletal maturity in the population was already confirmed in a previously published study.^16^

A previously published study evaluated the use of MRI to assess skeletal maturity at the knee by two paediatric radiologists and two general radiologists^26^ and found that the paediatric radiologists performed the task better than the general radiologists. Further, a recent study assessing the learning curve in screening mammogram interpretation^20^ showed that radiologists with more advanced training in breast imaging achieved a desirable performance for screening mammography in a shorter period of training compared to radiologists who did not undergo targeted breast radiology training. In this study, the blinded readers who achieved a high inter-reader agreement using a new MRI grading system of skeletal maturity were both board-certified paediatric radiologists. These findings underline the importance of targeted clinical specialty training when implementing a new radiological method for clinical use.

## Limitations

One limitation of the study was the use of a single cartilage-dedicated MRI sequence at a magnetic strength of 1.5 T. Multiple MRI sequences at 3 T might improve the resolution of the images and the evaluation of the growth plate grading. Different MRI scanners and vendors were used in the study based on the availability at the two facilities involved in the study. The use of different scanners might represent a limitation of the study, considering the relatively limited availability of MRI resources in clinical practice. On the other hand, the possibility of using the investigated MRI grading system of skeletal maturity on MRI different scanners and vendors shows the applicability of the MRI method for the purpose. The readers evaluating the MRI images in this study were board-certified paediatric radiologists. Assessment of the learning curves among general radiologists without targeted fellowship training in paediatric radiology might be valuable to examine the robustness of the investigated MRI grading system of skeletal maturity in less experienced readers.

## Conclusions

Paediatric radiologists’ clinical confidence when introduced to a new MRI grading system for skeletal maturity was high from the first time point of their learning curve, despite the radiologists having different levels of work experience and despite using the MRI grading system at five different growth plate locations. The investigated MRI grading system for skeletal maturity is a robust method when used by paediatric radiologists and can be used in clinical practice.

## Supplementary Material

tzae008_Supplementary_Data
