# Supplementary material for: Specialist learning curves and clinical feasibility of introducing a new MRI grading system for skeletal maturity
Source: BJR Open. 2024 Apr 10;6(1):tzae008. doi: 10.1093/bjro/tzae008 (PMC11052657; doi:10.1093/bjro/tzae008)
Supplement: tzae008_Supplementary_Data [file tzae008_supplementary_data.docx]

**Supplementary Table S1**. Detailed information about the MRI study protocols.

| **Vendor** | **Sequence** | **TE** | **TR** | **Flip angle** | **Wrist** | | | **Knee** | | | **Ankle** | | |
| --- | --- | --- | --- | --- | --- | --- | --- | --- | --- | --- | --- | --- | --- |
|  |  |  |  |  | **Matrix** | **FOV (mm)** | **Slice Thickness** | **Matrix** | **FOV** | **Slice Thickness** | **Matrix** | **FOV**  **(mm)** | **Slice Thickness** |
| GE  Signa | 3D-MERGE* | 18 ms | 40 ms | 5° | 208 x 208 | 120 x 120 | 2 mm | 272 x 218 | 160 x 160 | 3 mm | 300 x 161 | 150 x 150 | 3 mm |
| Philips  Achieva | 3DWATSc* | 7.6 ms | 20 ms | 25° | 208 x 208 | 120 x 120 | 2 mm | 272 x 218 | 160 x 160 | 3 mm | 300 x 161 | 150 x 150 | 3 mm |
| Siemens  Avanto Fit | MEDIC-3D* | 14 ms | 45 ms | 12° | 208 x 208 | 120 x 120 | 2 mm | 272 x 218 | 160 x 160 | 3 mm | 300 x 161 | 150 x 150 | 3 mm |
| *Fat saturation was included in all sequences  Wrist coil used: GE: Hi-Res Wrist MRI coil; Philips: PMS SENSE wrist coil; Siemens: 16 Ch Hand/wrist coil.  Knee coil used: GE: 8 Ch Knee T/R Knee coil; Philips: PMS SENSE 16 Ch Knee coil; Siemens: 15 Ch TxRx Knee coil.  Ankle coil used: GE: 8 Ch Ankle/foot coil; Philips: PMS SENSE foot/ankle array; Siemens: 16 Ch Foot/ankle coil. | | | | | | | | | | | | | |

**Supplementary Table S2. Modified version of Kellinghaus et al. (17) and Dedouit et al. (11) grading system.**

| **Stage 1** | Continuous, stripe-like, cartilage signal intensity is present between the metaphysis and the epiphysis with a thickness greater than 1.5mm with a multilaminar appearance.* |
| --- | --- |
| **Stage 2** | Continuous cartilage signal intensity is present between the metaphysis and the epiphysis with a thickness greater than 1.5 mm with increased signal intensity but without a multilaminar appearance.* |
| **Stage 3** | Continuous cartilage signal intensity is present between the metaphysis and the epiphysis with a thickness of less than 1.5mm with increased signal intensity.* |
| **Stage 4a** | The cartilage is not continuous. A blurred area involving one-third or less of the growth plate is present between the metaphysis and the epiphysis, representing epiphyseal-metaphyseal fusion.* |
| **Stage 4b** | The cartilage is not continuous. A blurred area involving between one-third and two-thirds of the growth plate is present between the metaphysis and the epiphysis, representing epiphyseal-metaphyseal fusion.* |
| **Stage 4c** | The cartilage is not continuous. A blurred area involving more than two-thirds of the growth plate is present between the metaphysis and the epiphysis, representing epiphyseal-metaphyseal fusion.* |
| **Stage 5** | The epiphyseal cartilage has fused completely, with or without an epiphyseal scar.† |
| *The image with the highest degree of closure was considered the most developed and was graded according to the modified staging system.  †Stage 5 grade required that the growth plate be completely closed on all images | |

**Supplementary Table S3.** Inter-reader reliability (R1 vs R2, mean and CI 95%) for non-cumulative time points.

| **Learning curve phase** | **1** |  | **2** |  | **3** |  | **4** |  | **5** |  |
| --- | --- | --- | --- | --- | --- | --- | --- | --- | --- | --- |
| **Distal radius** | ***Κ*** | **95% CI** | ***Κ*** | **95% CI** | ***Κ*** | **95% CI** | ***Κ*** | **95% CI** | ***Κ*** | **95% CI** |
| R1 vs R2 | .83 | .78-.88 | .72 | .66-.77 | .80 | .75-.84 | .89 | .84-.94 | .82 | .76-.89 |
| **Distal femur** |  |  |  |  |  |  |  |  |  |  |
| R1 vs R2 | .85 | .81-.90 | .72 | .66-.77 | .72 | .66-.77 | .76 | .70-.83 | .82 | .75-.89 |
| **Proximal tibia** |  |  |  |  |  |  |  |  |  |  |
| R1 vs R2 | .86 | .81-.91 | .74 | .68-.79 | .77 | .72-.83 | .82 | .76-.89 | .99 | .96-1.01 |
| **Distal tibia** |  |  |  |  |  |  |  |  |  |  |
| R1 vs R2 | .73 | .64-.83 | .78 | .72-.84 | .81 | .74-.88 | .85 | .77-.93 | .87 | .78-.96 |
| **Calcaneus** | | | | | | | | | | |
| R1 vs R2 | .80 | .70-.91 | .83 | .76-.90 | .92 | .85-.98 | .80 | .71-.90 | .90 | .81-.99 |
| **Overall mean kappa** | .81 |  | .76 |  | .80 |  | .82 |  | .88 |  |

***Κ***= Kappa value***,* 95% CI**= Confidence interval

**Supplementary Table S4.** Intra-reader reliability (R1 vs R1, mean and CI 95%) for non-cumulative time points.

| **Learning curve phase** | **1** |  | **2** |  | **3** |  | **4** |  | **5** |  |
| --- | --- | --- | --- | --- | --- | --- | --- | --- | --- | --- |
| **Distal radius** | ***Κ*** | **95% CI** | ***Κ*** | **95% CI** | ***Κ*** | **95% CI** | ***Κ*** | **95% CI** | ***Κ*** | **95% CI** |
| R1 vs R1 | .84 | .79-.90 | .74 | .68-.80 | .86 | .81-.91 | .94 | .91-.98 | .91 | .86-.97 |
| **Distal femur** |  |  |  |  |  |  |  |  |  |  |
| R1 vs R1 | .94 | .90-.97 | .77 | .71-.83 | .85 | .79-.90 | .83 | .76-.89 | .90 | .85-.95 |
| **Proximal tibia** |  |  |  |  |  |  |  |  |  |  |
| R1 vs R1 | .88 | .83-.93 | .83 | .78-.88 | .79 | .71-.87 | .93 | .88-.97 | .99 | .96-1.01 |
| **Distal tibia** |  |  |  |  |  |  |  |  |  |  |
| R1 vs R1 | .83 | .75-.91 | .83 | .77-.90 | .83 | .75-.91 | .94 | .88-.99 | .90 | .81-.98 |
| **Calcaneus** | | | | | | | | | | |
| R1 vs R1 | .88 | .81-.96 | .85 | .78-.93 | .95 | .90-1 | .88 | .79-.97 | .92 | .81-1.03 |
| **Overall mean kappa** | .87 |  | .80 |  | .86 |  | .90 |  | .92 |  |

***Κ***= Kappa value***,* 95% CI**= Confidence interval
